# Unique Triterpenoid of Jujube Root Protects Cisplatin-induced Damage in Kidney Epithelial LLC-PK1 Cells via Autophagy Regulation

**DOI:** 10.3390/nu12030677

**Published:** 2020-03-02

**Authors:** Dahae Lee, Kyo Bin Kang, Hyun Woo Kim, Jung Sik Park, Gwi Seo Hwang, Ki Sung Kang, Sungyoul Choi, Noriko Yamabe, Ki Hyun Kim

**Affiliations:** 1 School of Pharmacy, Sungkyunkwan University, Suwon 16419, Korea; pjsldh@naver.com; 2 Research Institute of Pharmaceutical Sciences, College of Pharmacy, Sookmyung Women’s University, Seoul 04310, Korea; kbkang@sookmyung.ac.kr; 3 Korea Bioactive Natural Material Bank, Research Institute of Pharmaceutical Sciences, College of Pharmacy, Seoul National University, Seoul 08826, Korea; kimkami2@snu.ac.kr; 4 College of Korean Medicine, Gachon University, Seongnam 13120, Korea; lucidpjs@naver.com (J.S.P.); seoul@gachon.ac.kr (G.S.H.); kkang@gachon.ac.kr (K.S.K.); pc1075@gachon.ac.kr (S.C.)

**Keywords:** cisplatin, nephrotoxicity, autophagy, *Ziziphus jujuba*

## Abstract

Chronic exposure to cisplatin is associated with irreversible kidney impairment. In this present study, we explored the protective effects of 3-dehydroxyceanothetric acid 2-methyl ester (3DC2ME) isolated from roots of jujube (*Ziziphus jujuba*, Rhamnaceae) against cisplatin-induced damage in vitro. In kidney epithelial LLC-PK1 cells, western blotting and staining with specific autophagy epifluorescent dye CytoID were used to determine the molecular pathways involving autophagy. Treatment with 3DC2ME reduced the increased Cyto-ID-stained autophagic vesicles and reversed the protein expressions of 5’ AMP-activated protein kinase subunit β-1 (AMPK)/mammalian target of rapamycin (mTOR)-dependent signaling pathway in cisplatin-induced cell death. Additionally, treatment with autophagy inhibitor 3-methyladenine (3-MA) and with or without 3DC2ME attenuated the cisplatin-induced apoptosis. Although further research is necessary to substantiate the effects, we evaluated the potential mechanism of action of 3DC2ME as an adjuvant for cancer patients.

## 1. Introduction

Cis-diamminedichloroplatinum (II), commonly known as cisplatin, is a chemotherapeutic anticancer drug. It remains one of the most effective anti-neoplastic drugs, owing to its broad-spectrum activity against a variety of cancers, with enhanced survival rate and life expectancy [1]. However, its use has been associated with numerous undesirable side-effects, including hematological toxicity, hepatic toxicity, nephrotoxicity, neurotoxicity, anorexia, and ototoxicity [2]. Among these, nephrotoxicity is considered to be a major concern as the kidneys are involved in the bio-transformation of various toxic chemicals, including anticancer drugs [3,4]. Moreover, cisplatin-induced toxicity resulted in a significant mortality rate of 50% to 60% [5]. These shortcomings have led to the development of novel therapeutic drugs and combination/synergistic strategies to overcome cisplatin-associated nephrotoxicity, without compromising its primary anticancer potential.

Historically, plant-derived compounds or phytochemicals have been widely used to treat various ailments such as cancer. Evidence in support of phytochemicals counteracting cisplatin-induced nephrotoxicity has started emerging [5,6]. A number of studies have recognized either whole or part of Jujube (*Ziziphus jujuba* Mill., Rhamnaceae) as an outstanding source of various phytochemicals, triterpenoids, polysaccharides, amino acids, flavonoids, alkaloids, polyphenols, and cerebrosides [7,8,9,10]. These compounds are known to exert several biological activities, including anti-oxidative [11,12,13,14], anti-inflammatory [13,15], anticancer [16,17], renoprotective [18,19], hepatoprotective [14,20], gastroprotective [21,22], neuroprotective [12], anti-obesity [23], anti-bacterial, and anti-diarrheal [24]. Among these compounds, triterpenoids are known to be abundantly present in all parts of *Z. jujube,* including fruits, leaves, roots, and seeds [10], and a further investigation into the effects of triterpenoids identified from roots of *Z. jujube* may uncover its other protective actions.

With this background, the present investigation characterized the protective effects of 3-dehydroxyceanothetric acid 2-methyl ester, a phytochemical isolated from roots of *Z. jujube*, in mitigating cisplatin-induced cell death in LLC-PK1. The LLC-PK1 is an established pig kidney epithelial cell line, with a characteristic of proximal epithelial cells that is commonly used for in vitro study of cisplatin-induced nephrotoxicity. This cell line is easily grown and maintained while retaining basic renal epithelial functions [25,26,27]. Its physiological and biochemical properties are similar to those of human renal proximal tubular cells, which allowed us to use it for nephrotoxicity studies [28].

Previous studies have suggested the positive or negative role of autophagy in preventing cisplatin-induced damage of renal cells [29]. Although its role in cisplatin-induced renal cell damage is not obvious, a low level of autophagy is important for maintaining the cellular homeostasis through the removal of damaged proteins and organelles that maintain cell survival. On the other hand, persistent autophagy involves apoptotic cell death [30]. There is growing evidence showing the negative role of autophagy in protecting renal cells from cisplatin-induced damage. A previous study has reported that ginsenoside Rb_3_ isolated from the leaves of *Panax quinquefolium* (American ginseng) inhibits cisplatin-induced cell death in HEK293 human embryonic kidney epithelial cells by regulating the 5’ AMP-activated protein kinase subunit β-1 (AMPK)/mammalian target of the rapamycin (mTOR)-dependent signaling pathway and apoptosis signaling pathway [31]. Recently, several studies have reported a relationship between autophagy and apoptosis in cisplatin-induced nephrotoxicity. Rapamycin (a specific inhibitor of mTOR) aggravates cisplatin-induced apoptosis in kidney epithelial cells [29,31], while ginsenoside Rb_3_ inhibits apoptosis via upregulation of phosphorylated mTOR and inhibition of cleaved caspase-3 in HEK293 cells [31]. Another previous study has shown that expression of cleaved caspase-3 is inhibited by autophagy inhibitor 3-methyladenine (3-MA) in NRK-52E rat renal proximal tubular cells, which demonstrates the positive role of 3-MA in protecting cells against cisplatin-induced apoptosis [29]. In this study, we investigated the protective effect of 3-dehydroxyceanothetric acid 2-methyl ester on the expression of autophagy-related proteins, including mTOR, 70-kDa ribosomal protein S6 kinase (p70S6K), AMPK, Beclin-1, and microtubule-associated protein light chain 3 (LC3) in the cisplatin-induced renal cell damage associated with apoptosis using autophagy inhibitor, 3-MA.

## 2. Materials and Methods

### 2.1. Preparation of 3DC2ME from Z. jujube

**Figure 1:**
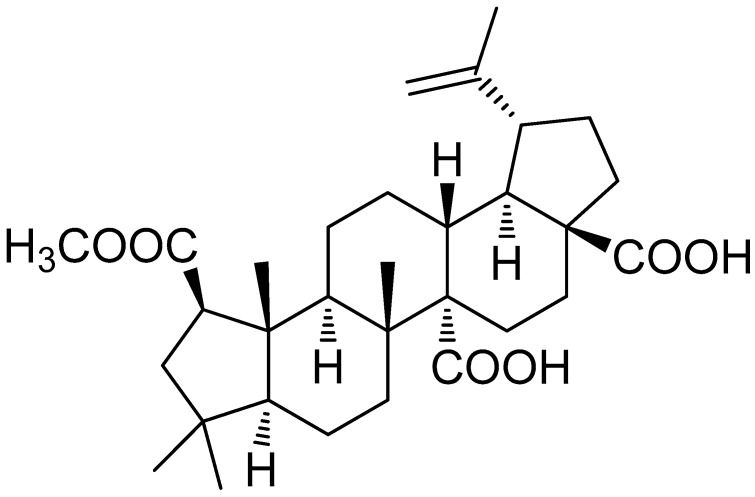
Chemical structure of 3DC2ME isolated from *Ziziphus jujuba*.

The isolation and chemical identification of 3-dehydroxyceanothetric acid 2-methyl ester (3DC2ME) are described in our previous study [32]. Briefly, the compound was isolated from the ethanolic extract of *Z. jujuba* roots using a series of column chromatography techniques. Chemical structure of isolated 3DC2ME was identified by nuclear magnetic resonance (NMR) spectroscopy (Figure 1) [32]. Its purity was determined to be above 90% from the NMR and liquid chromatography/mass spectrometry (LC/MS) analyses (Figures S1–S4).

### 2.2. Cell Culture and Reagents

Pig kidney epithelial cell line (LLC-PK1) was purchased from the American Type Culture Collection (ATCC, Manassas, VA, USA). LLC-PK1 cells were seeded into 100-mm dishes and grown in Dulbecco’s modified Eagle’s medium (DMEM) (ATCC, Manassas, VA, USA) containing 10% fetal bovine serum (Invitrogen, Grand Island, NY, USA) and 1% penicillin/streptomycin in an atmosphere of 5% CO_2_ at 37 °C. Cisplatin and 3-MA were purchased from Sigma-Aldrich (St. Louis, MO, USA). 

### 2.3. Protective Effect Against Cisplatin-Induced Nephrotoxicity in LLC-PK1 Kidney Cells

LLC-PK1 cells (1 × 10^4^ cells per well) were seeded into a 96-well plate for 24 h. Cells were pre-treated with varying concentrations of 3DC2ME and *N*-acetyl cysteine (NAC) served as the positive control for 2 h prior to treatment with cisplatin. The cell viability was measured using the Ez-Cytox cell proliferation/cytotoxicity assay kit. Following incubation for 24 h at 37 °C, 10 μL of Ez-Cytox (tetrazolium salts) reagent (Daeil Lab Services, Seoul, Korea) was added to each well. The cells were further incubated for 30 min at 37 °C, after which absorbance was measured using a microplate reader (PowerWave XS; Bio-Tek Instruments, Winooski, VT, USA) at 450 nM. 

### 2.4. Analysis of Autophagosomes

LLC-PK1 cells (2 × 10^5^ cells per well) were seeded into 8-well chamber slides or black 96-well clear flat-bottom plate. After treatment with 3DC2ME and/or cisplatin for different time intervals, cells on 8-well chamber slides were stained with specific autophagy epifluorescent dye CytoID (Enzo Life Sciences; Farmingdale, NY, USA) that detects autophagy vacuoles and Hoechst 33342 dye (Enzo Life Sciences; Farmingdale, NY, USA) for nuclear counterstain for 30 min in the dark at 37 °C, followed by 4% paraformaldehyde for 30 min in the dark at room temperature for fixation. Images were photographed using a fluorescent microscope (IX 50) equipped with a CCD camera. In addition, cells on a black 96-well clear flat-bottom plate were stained with specific autophagy epifluorescent dye CytoID for 30 min in the dark at 37 °C and washed with phosphate buffered saline (PBS). Intensity of Cyto-ID (green) fluorescence was measured using a fluorescent microplate reader (SPARK 10M) at 480/530 nM (ex/em). Data were expressed as fold changes compared with the untreated cells. 

### 2.5. Western Blot Analysis

LLC-PK1 cells (4 × 10^5^ cells per well) were seeded into a 6-well plate and then treated with 3DC2ME and/or cisplatin. After incubation for different time intervals, cells were lysed in radioimmunoprecipitation assay (RIPA) buffer (Cell Signaling Technology Inc., Beverly, MA, USA) containing with 1× ethylenediaminetetraacetic acid (EDTA)-free protease inhibitor cocktail and 1 mM phenylmethylsulfonyl fluoride (PMSF) based on the manufacturer’s instructions. After centrifugation at 2000 rpm for 20 min 4 °C, the supernatant was collected and total protein concentration was determined using the bicinchoninic acid (BCA) protein assay. Cell lysate containing 20 μg of protein was mixed with an equal volume of 4× sodium dodecyl sulfate (SDS) loading buffer and heated at 99 °C for 10 min. The lysate was loaded onto a 10% sodium dodecyl sulfate polyacrylamide gel electrophoresis (SDS–PAGE) for 90 min at 110 V along with molecular weight markers to separate different protein bands. After electrophoresis, the gel was then transferred to a polyvinylidene difluoride (PVDF) membrane for blocking. After blocking with 5% skim milk for 1 h at 4 °C, the membrane was incubated with primary antibodies against phospho-mTOR, mTOR, P-p70S6K, p70S6K, phospho-AMPKα, AMPKα, Beclin-1, LC3B, Bcl-2, Bax, cleaved caspase-3, and glyceraldehyde-3-phosphate dehydrogenase (GAPDH) (Cell Signaling Technology, Inc., Beverly, MA, USA) for 1 h at 4 °C. After washing, the membrane was then stained with horseradish peroxidase (HRP)-conjugated secondary antibodies for 1 h at 4 °C to detect primary antibodies. Imaging of protein bands was performed using enhanced chemiluminescence (ECL) Advance Western Blotting Detection Reagents (GE Healthcare, Cambridge, UK) and a FUSION Solo Chemiluminescence System (PEQLAB Biotechnologie GmbH, Erlangen, Germany).

### 2.6. Image-Based Cytometric Assay

LLC-PK1 cells (4 × 10^5^ cells per well) were seeded into a 6-well plate and then treated with 3DC2ME and/or cisplatin. After incubation for different time intervals, cells were harvested and resuspended in binding buffer (Life Technologies, Carlsbad, CA, USA). After centrifugation at 1000 rpm for 5 min at 37 °C, the supernatant was removed, and cells were stained with 5 μL annexin V Alexa Fluor 488 (Invitrogen, Temecula, CA, USA) for 30 min in the dark. Annexin V-positive-stained apoptotic cells were counted via a Tali image-based cytometer (Invitrogen, Temecula, CA, USA).

### 2.7. Statistical Analysis

Proliferation/cytotoxicity assay, analysis of autophagosomes, and western blot analysis were done simultaneously for each assay and were repeated at least four times. All data are shown as means ± standard deviation (SD). Analysis of variance (ANOVA) was used for statistical significance with post hoc Bonferroni multiple testing correction and hypergeometric tests. A *p*-value less than 0.05 was considered as statistically significant.

## 3. Results

### 3.1. Protective Effects of 3DC2ME Against Cisplatin-Induced Kidney Cell Damage

**Figure 2:**
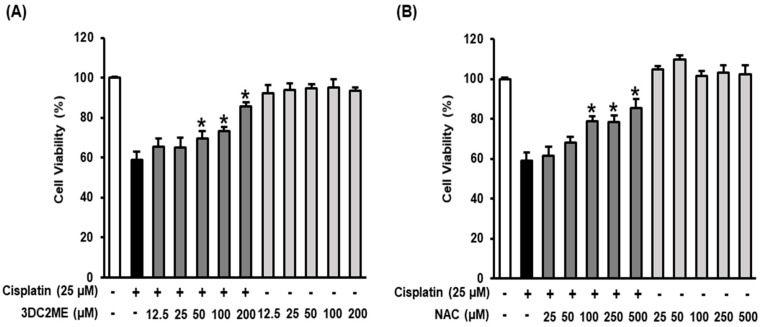
Protective effect of 3DC2ME isolated from *Z. jujuba* root extract against cisplatin-induced kidney cell damage. (**A**) Effects of 3DC2ME and (**B**) NAC on viability of LLC-PK1 cells exposed to 25 μM cisplatin for 24 h using the Ez-Cytox cell viability assay (mean ± SD, * *p* < 0.05 cisplatin-treated LLC-PK1 cells). 3DC2ME, 3-dehydroxyceanothetric acid 2-methyl ester; NAC, *N*-acetyl cysteine; SD, standard deviation.

To analyze the protective effects of 3DC2ME isolated from *Z. jujuba* root extract, LLC-PK1 cells were pre-treated with varying concentrations of 3DC2ME for 2 h (12.5, 25, 50, 100, and 200 μM) and then, treated with 25 μM cisplatin for 24 h. *N*-acetyl cysteine (NAC) was used as the positive control. The cell viability was then measured using the Ez-Cytox cell viability assay. As shown in Figure 2A, treatment with cisplatin alone resulted in cell viability of 58.96% ± 3.96%. Cell viability was increased to 85.53% ± 2.16% and 85.49% ± 4.29% by 200 μM 3DC2ME and 500 μM NAC, respectively. These results indicated that 3DC2ME showed a better protective effect at a lower concentration than NAC (positive control). When treated with 3DC2ME alone, the cell viability was over 90% at all concentrations. Accordingly, we used 100 and 200 μM of 3DC2ME for further experiments.

### 3.2. Effect of 3DC2ME on Autophagic Vacuoles in LLC-PK1 Cells

**Figure 3:**
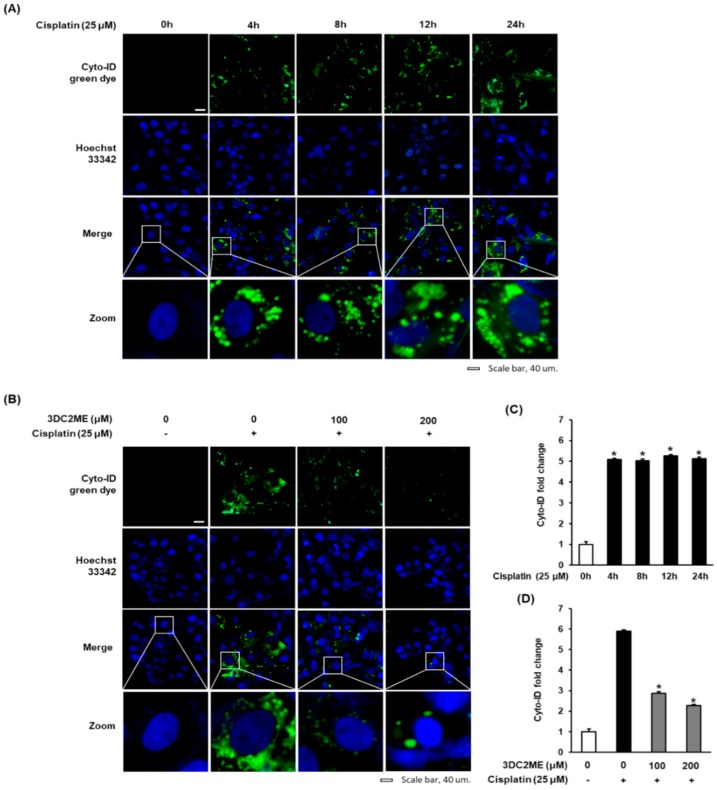
Effect of 3DC2ME isolated from *Z. jujuba* root extract on autophagic vacuoles in LLC-PK1 cells. (**A**) Representative images of autophagic LLC-PK1 cells stained with Cyto-ID (**green**) at various time points as indicated and exposed to 25 μM cisplatin. Nuclei are counterstained with Hoechst 33342 dye (**blue**). (**B**) Representative images of autophagic LLC-PK1 cells exposed to 3DC2ME in the presence of 25 μM cisplatin after staining with Cyto-ID (green) for 24 h. Nuclei were counterstained with Hoechst 33342 dye (**blue**). (**C**,**D**) Bar graphs indicated the fold of Cyto-ID (**green**) fluorescence intensity in each group as compared with control cells. Scale bar, 40 μm (mean ± SD, * *p* < 0.05 cisplatin-treated LLC-PK1 cells). 3DC2ME, 3-dehydroxyceanothetric acid 2-methyl ester; SD, standard deviation.

To determine the potential of cisplatin to induce autophagy in LLC-PK1 cells, a Cyto-ID autophagy detection kit was used. LLC-PK1 cells were exposed to 25 μM cisplatin and stained with Cyto-ID autophagy detection dye at time points 0, 4, 8, 12, and 24 h. As shown in Figure 3A, fluorescent microscopy showed a gradual increase in Cyto-ID green fluorescence in a time-dependent manner with maximum effect at 24 h. Consistent with this result, quantitation of the results in Figure 3C showed that treatment with 25 µM cisplatin for 4, 8, 12, and 24 h resulted in 5.08 ± 0.05-fold, 5.02 ± 0.08-fold, 5.25 ± 0.06-fold and 5.12 ± 0.08-fold increase in Cyto-ID green fluorescence fold change. We then evaluated the effects of 3DC2ME (100 and 200 μM) on autophagy in LLC-PK1 cells at 24 h. As shown in Figure 3B, LLC-PK1 cells treated with 25 μM cisplatin exhibited a significant increase in Cyto-ID green fluorescence, whereas treatment with 100 and 200 μM 3DC2ME reduced the signal in a concentration-dependent manner. Consistent with this result, quantitation of the results in Figure 3D showed that, after treatment of 25 µM cisplatin for 24 h, the Cyto-ID green fluorescence fold change was significantly increased to 5.90 ± 0.03-fold, whereas treatment with 100 or 200 µM 3DC2ME decreased Cyto-ID green fluorescence fold change to 2.87 ± 0.02-fold and 2.26 ± 0.15-fold, respectively. These results suggested that 3DC2ME prevented cisplatin-induced cell death through autophagy inhibition.

### 3.3. Effect of Cisplatin on Protein Expressions of AMPK/mTOR-Dependent Signaling Pathway in LLC-PK1 Cells

**Figure 4:**
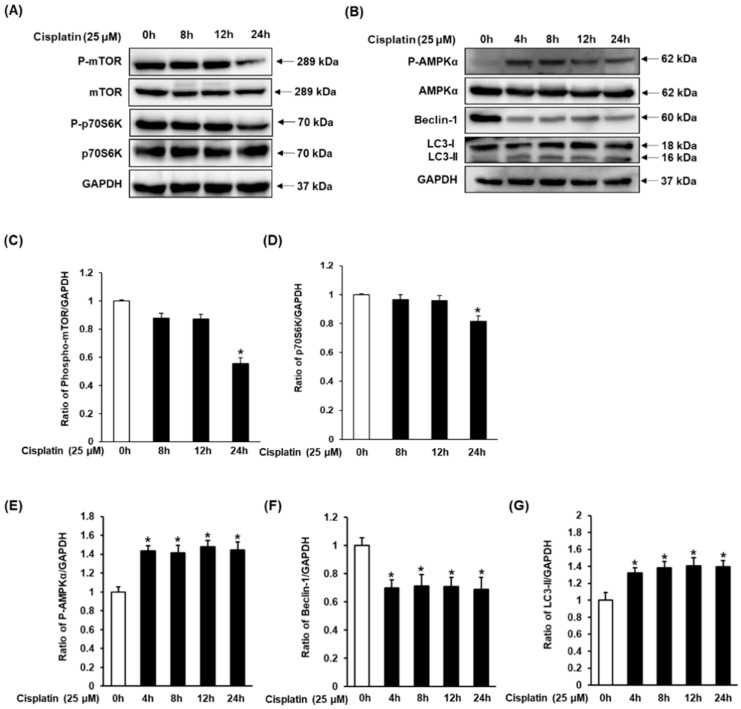
Time-course protein expressions of (**A**) phospho-mTOR, mTOR, phospho-p70S6K, p70S6K, and (**B**) phospho-AMPKα, AMPKα, Beclin-1, and LC3 in LLC-PK1 cells exposed to 25 μM cisplatin by western blotting. (**C**–**G**) Bar graphs indicated the relative ratio of the western blot band signals in each group as compared with control cells (mean ± SD, * *p* < 0.05 cisplatin-treated LLC-PK1 cells). SD, standard deviation.

To evaluate the effect of cisplatin on protein expressions of AMPK/mTOR-dependent signaling pathway, LLC-PK1 cells were exposed to 25 μM cisplatin and harvested at time points 0, 4, 8, 12, and 24 h. As shown in Figure 4A, decreased expression of phospho-mTOR and phospho-p70S6K was detected at 24 h as compared to their expression at 0 h. As shown in Figure 4B, increased expression of phospho-AMPKα and LC3, and decreased expression of Beclin-1 were identified at all time-points in comparison to their expression at 0 h. Phospho-AMPKα and Beclin-1 did not show a time-dependent expression. Contrary to this, expression of LC3 gradually increased in a time-dependent manner, reaching its maximum at 24 h. 

### 3.4. Effect of 3DC2ME on Protein Expressions of AMPK/mTOR-Dependent Signaling Pathway in LLC-PK1 Cells

**Figure 5:**
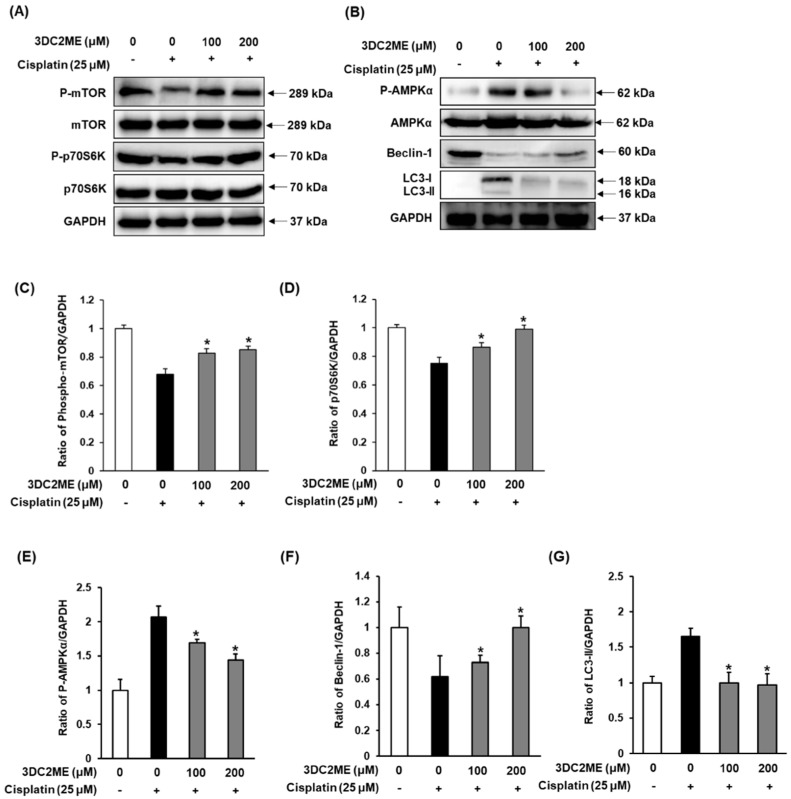
Effect of 3DC2ME isolated from *Z. jujuba* root extract on expression of (**A**) phospho-mTOR, mTOR, phospho-p70S6K, p70S6K, and (**B**) phospho-AMPKα, AMPKα, Beclin-1, and LC3 in LLC-PK1 cells exposed to 25 μM cisplatin by western blotting. (**C**–**G**) Bar graphs indicated the relative ratio of the western blot band signals in each group as compared with control cells.(mean ± SD, * *p* < 0.05 cisplatin-treated LLC-PK1 cells). 3DC2ME, 3-dehydroxyceanothetric acid 2-methyl ester; 3-MA, 3-methyladenine; SD, standard deviation.

Next, we evaluated the effects of 3DC2ME on the protein expressions associated with autophagy pathways by western blotting. Results were analyzed from LLC-PK1 cells treated with 25 μM cisplatin for 24 h with and without 3DC2ME (100 and 200 μM). As shown in Figure 5A,B, increased expression of phospho-AMPKα and LC3, and decreased expression of Beclin-1, phospho-mTOR, phospho-p70S6K were detected in LLC-PK1 cells exposed to 25 μM cisplatin at 24 h as compared with control cells. Contrary to this, exposure to 100 and 200 μM 3DC2ME reversed the activation of protein expressions of AMPK/mTOR-dependent signaling pathway, completely.

### 3.5. Effects of Autophagy Inhibitor 3-MA and 3DC2ME on Expression of Autophagy-Related Protein and Apoptosis-Related Proteins in LLC-PK1 Cells

**Figure 6:**
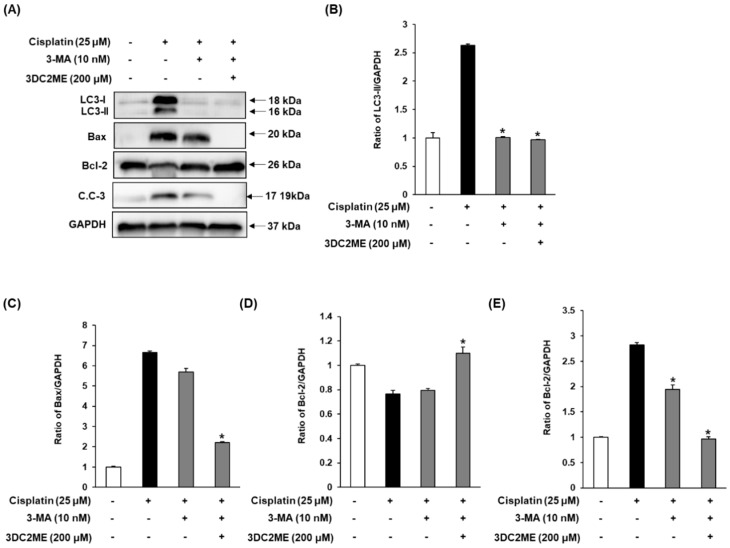
Effect of autophagy inhibitor 3-MA and 3DC2ME isolated from *Z. jujuba* root extract on expression of (**A**) LC3, Bax, Bcl-2 and cleaved caspase-3 in LLC-PK1 cells treated with 25 μM cisplatin by western blotting in the presence or absence of 3-MA. (**B**–**E**) Bar graphs indicated the relative ratio of the western blot band signals in each group as compared with control cells (mean ± SD, * *p* < 0.05 cisplatin-treated LLC-PK1 cells). 3DC2ME, 3-dehydroxyceanothetric acid 2-methyl ester; 3-MA, 3-methyladenine; SD, standard deviation.

Exposure to 25 μM cisplatin leads to increased LC3, Bax/Bcl-2 ratio and cleaved caspase-3 (Figure 6A). Contrary to this, exposure to 10 nM 3-MA and with or without 200 μM 3DC2ME completely reversed the activation and expression of autophagy- and apoptosis-related proteins.

### 3.6. Effects of Autophagy Inhibitor 3-MA and 3DC2ME Against Cisplatin-Induced Apoptosis in LLC-PK1 Cells

**Figure 7:**
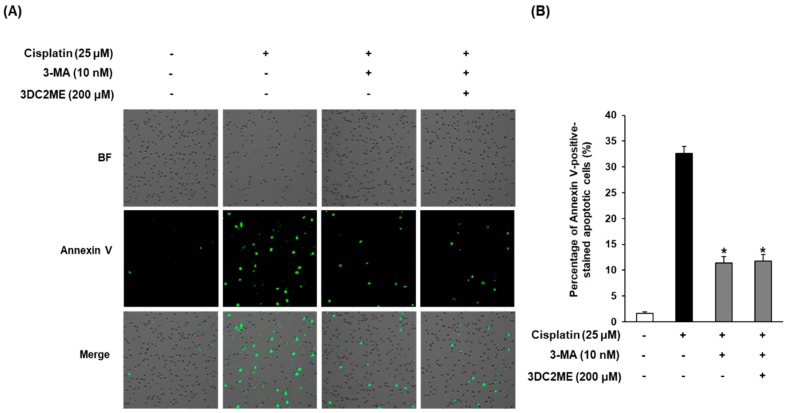
Reduction of apoptosis after treatment of autophagy inhibitor 3-MA and 3DC2ME in LLC-PK1 cells exposed to 25 μM cisplatin for 24 h. (**A**) Images acquired for quantitative measures of apoptosis and (**B**) percentage of annexin V-positive-stained apoptotic cells. Control cells were treated with vehicle only (mean ± SD, * *p* < 0.05 compared to control). 3-MA, 3-methyladenine; SD, standard deviation.

Lastly, we explored whether 10 nM 3-MA and 200 μM 3DC2ME could reduce cisplatin-induced apoptosis in LLC-PK1 cells. As shown in Figure 7, after exposure to 25 μM cisplatin, the percentage of annexin V-positive cells, indicative of apoptosis, was increased to 32.6% ± 1.41%, whereas it was decreased by treatment with 10 nM 3-MA and with or without 200 μM 3DC2ME to 11.80% ± 1.24% or 11.43% ± 1.20%, respectively.

## 4. Discussion

In our previous study, we evaluated the effects of 3DC2ME, a triterpenoid isolated from *Z. jujuba* root extract, in overcoming cisplatin-induced kidney cell damage. We observed that 3DC2ME efficiently prevents cisplatin-induced renal toxicity via modulating MAPK and apoptosis pathways [33]. The present study added further mechanism study that 3DC2ME efficiently protects from cisplatin-induced renal cell toxicity. We separated four fractions (CHCl_3_, EtOAc, *n*-BuOH, and H_2_O) from *Z. jujuba* root extract and evaluated them for their ability to counteract cisplatin-induced kidney cell damage. All fractions could significantly reverse the cisplatin-induced decrease in LLC-PK1 cell viability (Figure S5) when used in combination with cisplatin. In particular, CHCl_3_ fraction exerted the highest protective effect against cisplatin-mediated renal toxicity (Figure S5). We further confirmed the protective effect of 3DC2ME by comparing its beneficial effect with that of NAC (used as the positive control), and the results proved it to exert better protective action than NAC. 

Numerous reports have demonstrated the protective ability of triterpenoids, isolated from various natural products, such as arjunolic acid isolated from *Terminalia arjuna* [34,35] and ginsenosides Rk_3_ and Rh_4_ isolated from heat-processed *Panax ginseng* [36], and lupane triterpenes isolated from *Cornus walteri* [37]; these have proven to be effective in attenuating cisplatin-induced nephrotoxicity. These phytochemicals exerted their protective effects via modulating the pathophysiology of drugs, including oxidative stress [34,35], inflammation [36], and apoptosis [36,37]. Studies have also demonstrated autophagic pathways to trigger cisplatin-associated kidney cell damage [38,39]. However, autophagy-related signaling pathways in cisplatin-induced kidney cell damage has remained elusive. Autophagy may exert an either protective or lethal effect, depending on the experimental situation [40,41]. For example, some studies have suggested inhibition of autophagy to induce cisplatin-induced kidney cell damage [39,42], whereas other studies have shown autophagy to be a contributing factor [43,44]. Autophagy is a regulatory process induced in cells in response to stress stimuli; in certain circumstances, it may also lead to apoptosis or necrosis [43]. Cisplatin-induced cell damage in LLC-PK1 cells was accompanied by a time-dependent gradual increase in Cyto-ID-stained autophagic vesicles. Interestingly, treatment with 100 and 200 μM 3DC2ME reduced the increased Cyto-ID-stained autophagic vesicles in a concentration-dependent manner. 

Autophagy has been known to be both directly and indirectly activated by AMPK that inhibits mTOR, involved in autophagy as an important negative regulator [31,38]. AMPK phosphorylation also directly regulates Beclin-1 [45]. Cisplatin-induced autophagy in kidney epithelial cells was associated with an increase in phosphorylation of AMPK and decrease in phosphorylation of mTOR and its downstream target p70S6K [31]. Additionally, LC3, another protein involved in autophagy, is post-translationally modified from its cytosolic form (LC3-I) to active membrane-bound form (LC3-II) during autophagy. This results in a specific association of LC3-II with autophagosome formation, making it a biomarker of autophagy [39,46]. 

We, therefore, focused on evaluating the effect of 3DC2ME on expression of mTOR, p70S6K, AMPKα, Beclin-1, and LC3. The significant increase in the levels of phosphorylated AMPK just 4 h of cisplatin treatment, which gradually increased until 24 h, has also been previously reported [47]. Similarly, levels of Beclin-1 were found to decrease after 4 h of cisplatin treatment; the levels gradually decreased until 24 h, consistent with a previous observation [39]. The observation that these changes in the levels of phosphorylated AMPKα and Beclin-1 could be reversed following treatment with 3DC2ME is evident of the involvement of autophagy proteins in counteracting cisplatin-mediated nephrotoxicity. A previous study has shown that cisplatin can lead to a decrease in the expression level of mTOR in HEK293 cells [31]. Although the cell types are different, our results are similar in that decreased expression of phospho-mTOR and phospho-p70S6K were detected in LLC-PK1 cells exposed to 25 μM cisplatin at 24 h as compared with control cells, while 3DC2ME enhanced the expression of phospho-mTOR and phospho-p70S6K. The present study also reported induction of LC3-I and its conversion to LC3-II after 4 h of cisplatin treatment, with a gradual increase until 24 h. Treatment with 3DC2ME reversed these effects. Although not all experiments related to autophagy were performed, it was confirmed that some of the mechanisms of action of 3DC2ME were involved. This autophagy activity was significantly inhibited by 3DC2ME via the AMPK/mTOR-dependent signaling pathway, strengthening the crucial role of phytochemicals in alleviating cisplatin-induced toxicity.

To examine the involvement of autophagy in the apoptotic pathway focusing on the role of Bcl-2, Bax, and caspase 3 proteins, we used 3-MA. Western blot analysis of LC3 as an autophagy-related protein and Bcl-2, Bax, cleaved caspase 3 as an apoptosis-related protein were performed. In our previous study, treatment with 3DC2ME significantly attenuated the increase in cisplatin-induced apoptosis through the downregulation of pro-apoptotic Bax, cleaved caspase-3, which is a critical factor in the execution of apoptosis and upregulation of anti-apoptotic Bcl-2 [33]. It was also shown in the current study that in the absence of cisplatin, levels of LC3, Bax/Bcl-2 ratio, and cleaved caspase-3 were increased, but these levels were significantly depressed by 3-MA and/or 3DC2ME. Furthermore, the percentage of annexin V-positive cells indicating apoptosis was increased by treatment with cisplatin, whereas it was suppressed by treatment with 3-MA and with or without 3DC2ME. Overall, these data suggested that inhibition of autophagy might attenuate cisplatin-induced apoptosis in LLC-PK1 cells. 

This study demonstrated that, for the first time, 3DC2ME is associated with autophagy regulation in overcoming cisplatin-induced kidney cell death, which verifies the beneficial protective role of 3DC2ME. Moreover, the autophagy regulation aided in the cellular protective effect of 3DC2ME, preventing kidney cells from cisplatin-induced apoptosis. Therefore, further studies discerning the relationship between autophagy and apoptosis, along with factors related to cisplatin-induced cytotoxicity, are suggested to explore the potential clinical application of 3DC2ME.

## 5. Conclusions

In conclusion, results of the present findings indicated that 3DC2ME, a triterpenoid isolated from *Z. jujube*, exerts protective effects against cisplatin-induced death in LLC-PK1 cells. Inhibition of the AMPK/mTOR-dependent signaling pathway involved in autophagy regulation enhanced the cellular protective effect of 3DC2ME from cisplatin-induced apoptosis. Further studies on the analysis of additional factors related to cisplatin-induced cytotoxicity are needed to clarify mechanisms involved in 3DC2ME action that could help to develop novel therapeutic strategies for cancer patients with kidney damage. 

## Supplementary Materials

The following are available online at https://www.mdpi.com/2072-6643/12/3/677/s1, Figure S1: ^1^H NMR spectrum of 3DC2ME. Figure S2: ^13^C NMR spectrum of 3DC2ME. Figure S3: HRESIMS of 3DC2ME. Figure S4: LC/MS analysis of 3DC2ME. Figure S5: Protective effect of *Z. jujuba* root extract and its four fractions against cisplatin-induced kidney cell damage.
