# Supplementary material for: Unique Triterpenoid of Jujube Root Protects Cisplatin-induced Damage in Kidney Epithelial LLC-PK1 Cells via Autophagy Regulation
Source: Nutrients. 2020 Mar 2;12(3):677. doi: 10.3390/nu12030677 (PMC7146250; doi:10.3390/nu12030677)
Supplement: Supplementary file 1 [file nutrients-12-00677-s001.pdf]

Contents

Materials and Methods……………………………………………………………..…………3

Characterization data of 3DC2ME ..…………………………………………………4

Figure S1. ^{1}H NMR spectrum of 3DC2ME..5

Figure S2. ^{13}C NMR spectrum of 3DC2ME..5

Figure S3. HRESIMS of 3DC2ME…………………………………………………..6

Figure S4. LC/MS analysis of 3DC2ME……………………………………………..6

Figure S5. Protective effect of *Z. jujuba* root extract and its four fractions against cisplatin-induced kidney cell damage………………………………………………………………7

Materials and Methods

Preparation of fractions and 3DC2ME from Ziziphus jujuba

The roots of *Z. jujuba* were collected in April 2012 at Jinju, Korea and authenticated by Prof. Dr. Eun Ju Jeong (Gyeongnam National University of Science and Technology, Jinju, Korea). A voucher specimen (SUPH-1204-01) was deposited in the Herbarium of the Medicinal Plant Garden, College of Pharmacy, Seoul National University, Koyang, Korea. Pulverized, air-dried roots of *Z. jujuba* (7.5 kg) were extracted with EtOH (2 × 30 L, for 3 h each) with ultrasonication at room temperature and then concentrated *in vacuo*. The crude extract (630.4 g) was suspended in H_2O and partitioned successively into $CHCl_3$ (103.5 g), EtOAc (75.0 g), and *n*-BuOH fractions (127.3 g), respectively. The $CHCl_3$ fraction was subjected to silica gel column chromatography (CC) eluted with mixtures of $CHCl_3$-MeOH (100:1, 50:1, 25:1, 15:1, 10:1, 7:1, 5:1, and 3:1) to yield ten fractions (C1–C10). The MeOH-soluble part of fraction C5 was subjected to silica gel CC eluted with $CHCl_3$-MeOH mixtures of increasing polarity (100:1, 50:1, 25:1, 15:1, 10:1) to give ten subfractions (C5a–C5j). Subfraction C5e was separated into seven further subfractions (C5e1–C5e7) by silica gel CC with mixtures of $CHCl_3$-MeOH of increasing polarity (100:1, 50:1, 25:1, 15:1, 10:1). White pellets of subfraction C5e2, which were insoluble in MeOH, were filtered and purified by recrystallization with MeOH to yield 3-dehydroxyceanothetric acid 2-methyl ester (3DC2ME) (45.8 mg).

Characterization data

3-Dehydroxyceanothetric Acid 2-Methyl Ester.

White amorphous powder; mp 296–298 °C; $[\alpha]_D^{20}$ +73.4 (*c* 0.10, MeOH); IR ν_{max} 3704, 2950, 2869, 2361, 2327, 1687, 1054, 1033, 1013 cm^{-1}; ^1H NMR (600 MHz, pyridine-d_5) δ 5.08 (s, 1H, H-29a), 4.82 (s, 1H, H-29b), 3.78 (s, -OCH_3), 3.71 (m, 1H, H-19), 2.98 (dt, 1H, *J* = 4.8, 12.9 Hz, H-13), 2.69 (d, 1H, *J* = 7.6 Hz), 1.92 (s, 3H, H-30), 1.17 (s, 3H, H-23), 1.16 (s, 3H, H-26), 0.89 (s, 3H, H-24), 0.89 (s, 3H, H-25); ^{13}C NMR (150 MHz, pyridine-d_5) δ 178.9 (C-27), 179.8 (C-28), 176.8 (C-2), 151.6 (C-20), 110.7 (C-29), 60.8 (C-14), 57.0 (C-17), 56.5 (C-5), 55.7 (C-1), 52.7 (C-18), 51.6 (C-10), 48. 3(C-19), 46.5 (C-9), 42.6 (C-3), 41.7 (C-8), 40.7 (C-13), 38.6 (C-4), 38.1 (C-22), 38.1 (C-7), 35.8 (C-16), 31.9 (C-23), 31.6 (C-21), 29.3 (C-15), 27.2 (C-12), 27.0 (C-24), 24.4 (C-11), 19.8 (C-25), 19.8 (C-30), 19.1 (C-6), 18.5 (C-26); HRESIMS *m/z* 513.3208 $[M - H]^-$ (calcd for $C_{31}H_{45}O_6$, 513.3216).

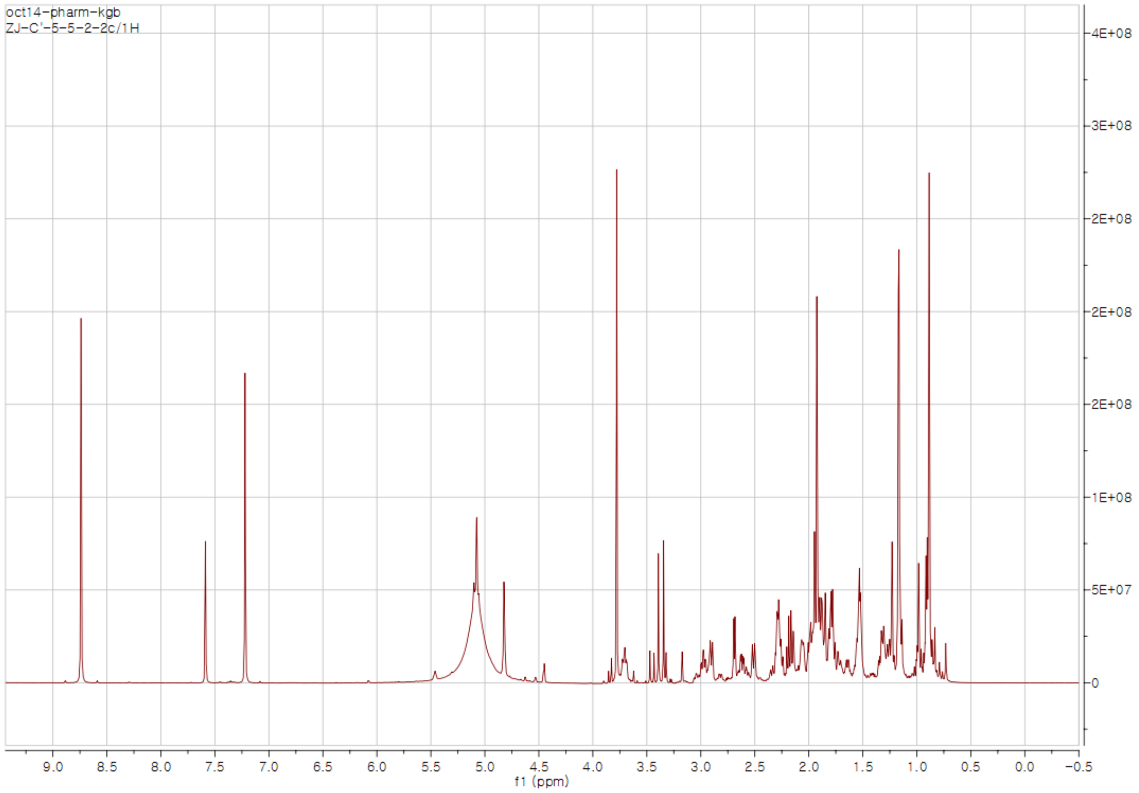

Figure S1. ^{1}H NMR spectrum of 3DC2ME.

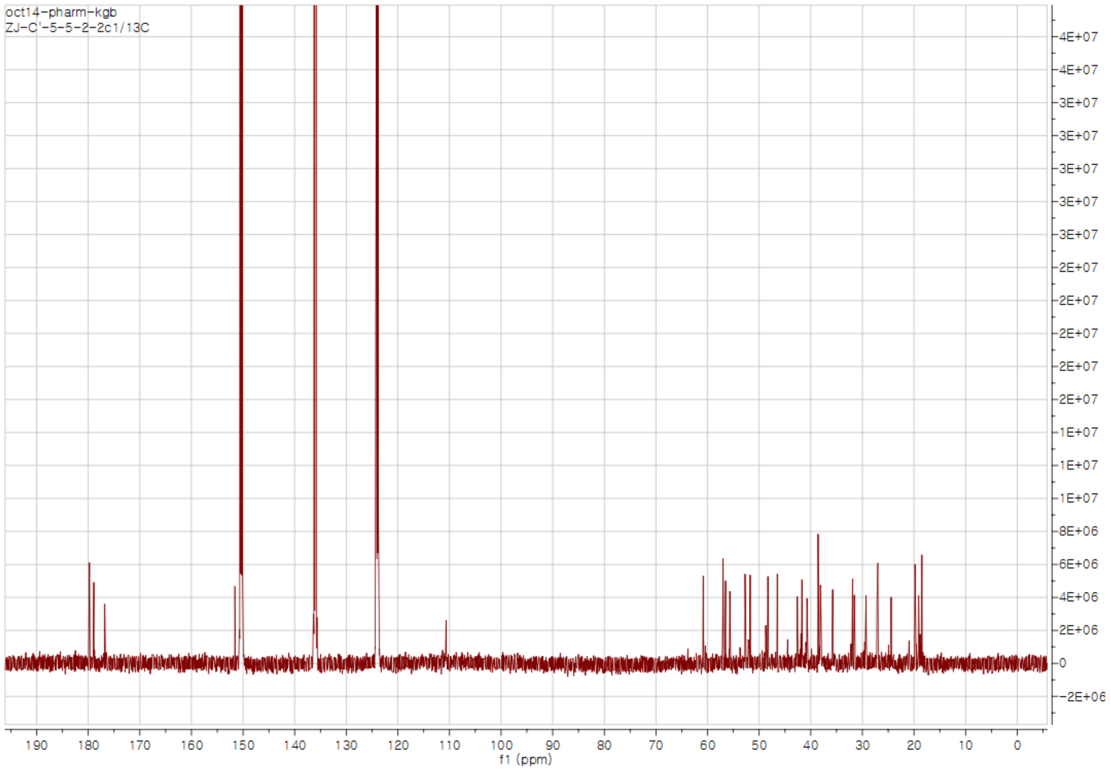

Figure S2. ^{13}C NMR spectrum of 3DC2ME.

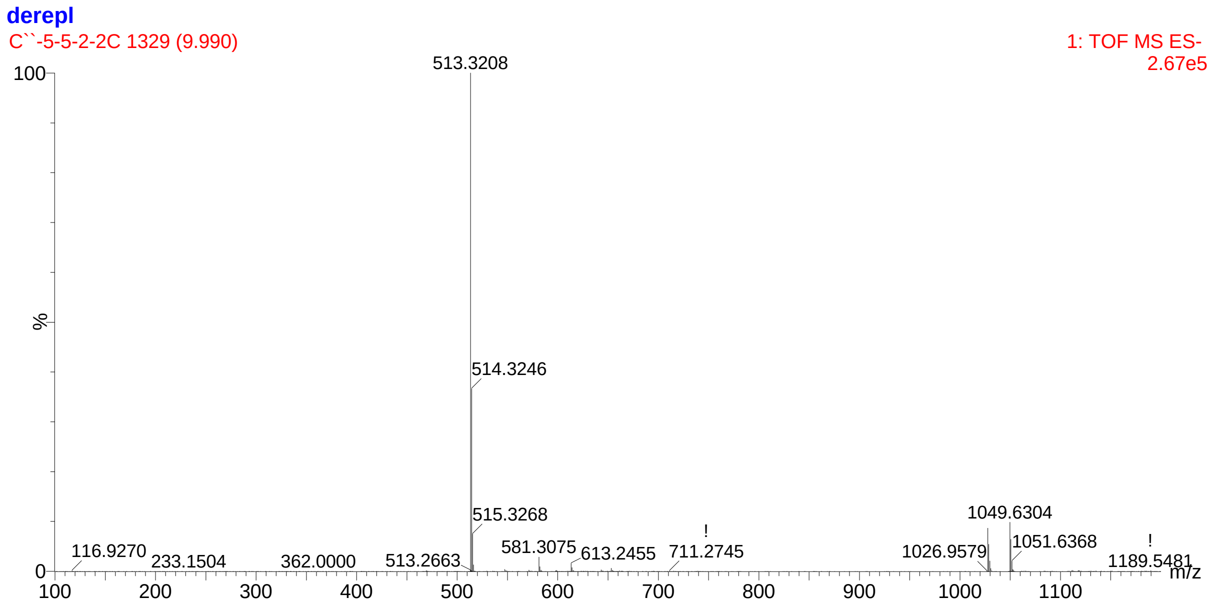

Figure S3. HRESIMS of 3DC2ME.

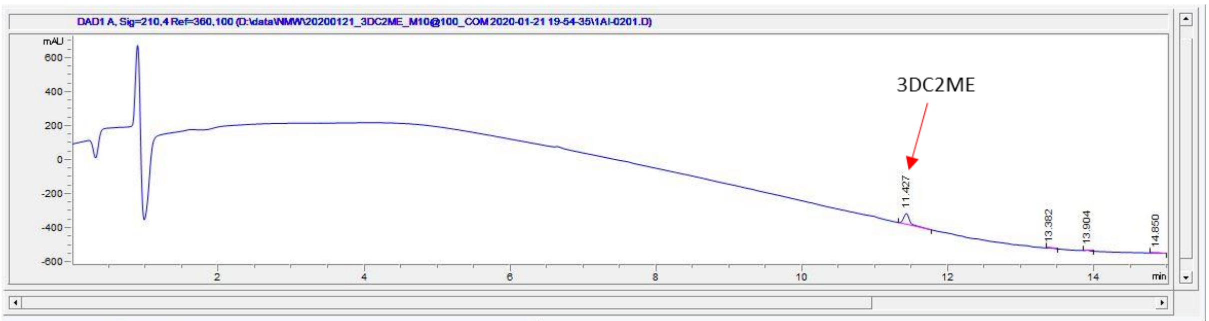

File Information

LC-File	1AI-0201.D
File Path	D:\data\NMW\20200121_3DC2ME_M10@100_COM 2020-01-21 19-
Date	21-Jan-20, 20:16:38
Sample	1
Sample Info	
Barcode	
Operator	SYSTEM
Method	10%~100%MeOH_COMPOUNDS(ms100@1200).M

#	Time	Area	Height	Width	Area%	Symmetry
1	11.427	361.7	62.1	0.088	91.738	0.726
2	13.382	12.6	1.8	0.0967	3.196	0.171
3	13.904	10.4	3.5	0.0489	2.627	0.64
4	14.85	9.6	1.7	0.0843	2.439	0.586

Figure S4. LC/MS analysis of 3DC2ME [detection wavelength was set at 210 nm; The mobile phase consisted of H_2O (A) and MeOH (B) with a gradient system as follows: 10-100% B (0-15 min); flow rate = 0.3 mL/min; analytical Kinetex (4.6 × 100 mm, 3.5 μm) column].

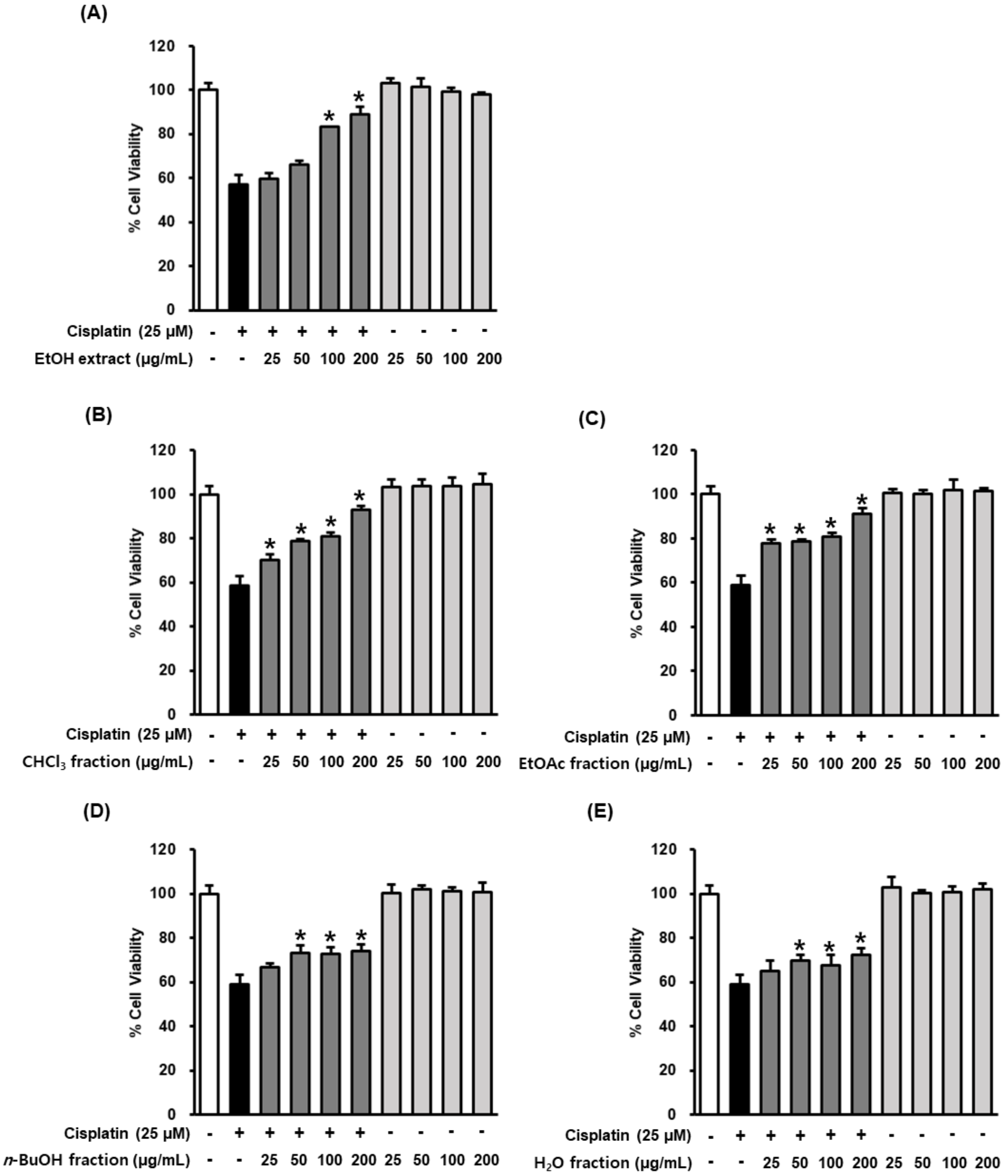

Figure S5. Protective effect of *Z. jujuba* root extract and its four fractions against cisplatin-induced kidney cell damage. Effects of (A) EtOH extract and (B) $CHCl_3$, (C) EtOAc, (D) *n*-BuOH, (E) H_2O fractions on viability LLC-PK1 cells exposed to 25 μM cisplatin for 24 h using the Ez-Cytox cell viability assay. (mean ± SD, * $p < 0.05$ cisplatin-treated LLC-PK1 cells). EtOH, ethyl alcohol; $CHCl_3$, chloroform; EtOAc, ethyl acetate; *n*-BuOH, *n*-butanol; H_2O, water; SD, standard deviation.
